# Factors associated with health CEO turnover - a scoping review

**DOI:** 10.1186/s12913-024-11246-y

**Published:** 2024-07-29

**Authors:** Nebu Varughese Mathew, Chaojie Liu, Hanan Khalil

**Affiliations:** La Trobe University, Melbourne, VIC 3086 Australia

**Keywords:** CEO, Chief executive officer, Top executive, Turnover, Turnover rates, Replacement, Retention, Healthcare, Firms

## Abstract

**Background:**

Chief Executive Officer’s (CEO) are integral leaders of health care organisation. Over the last two decades in United States (US) hospitals, it has been noted that CEO turnover rates are increasing, and it was reported that the CEO turnover rates have augmented from 14% in 2008 to 18% in 2017 in the private sector. In Australia, it was discovered that during two years, 41 executives had 18 distinct positions. It has been highlighted that the increasing CEO turnover is a major issue for Australian and international health care organisations. Some of the negative consequences of CEO turnover include organisational instability, high financial costs, loss of human capital and adverse effects on staff morale and patient care.

**Objective:**

Our scoping review aimed to map and summarise the evidence associated with CEO turnovers in both health and non-health setting, and answer the following questions: 1. What are the reasons for CEO departure?, 2. What are the strategies to minimise CEO turnover?

**Results:**

A protocol explaining the objectives, inclusion criteria and methods for this scoping review were specified in advance and published. This scoping review included 17 studies (13 health and 4 non-health setting) published over a 31-year period that investigated and described the increasing CEO turnover rates. All 17 studies identified causes of CEO turnover along with certain studies identifying facilitators of CEO retention. We classified CEO’s departure reasons into three major themes: organizational, performance, and personal. Organisational factors include CEO departures due to issues within the organisation, performance factors include issues with CEO’s work and personal factors captures personal reasons for CEO’s leaving their job.

**Conclusion:**

CEOs are under immense pressure to deliver good results and drive growth while satisfying the interests of internal and external stakeholders. There are various reasons for CEO’s departure however the most common factor identified is organisational.

**Supplementary Information:**

The online version contains supplementary material available at 10.1186/s12913-024-11246-y.

## Contribution of paper statement

**What is already known**

Causes and consequences of CEO turnover in both health and non-health settings.

**What this paper adds**

Three main factors associated with CEO turnover such as organisational, performance and personal factors.

**Supplementary Information:**

The online version contains supplementary material available at 10.1186/s12913-024-11246-y.

## Background

A Chief Executive Officer (CEO) is a top rank executive in an organisation who manages daily operations, makes corporate decisions and is the public face of the organisation [1]. With changing economies, CEOs are finding their job to be increasingly difficult as they must manage tight budgets, model a positive work culture, manage daily operations and follow the Board’s initiatives and strategic plans. The CEO role demands significant time, effort and critical thinking which makes the job one of the busiest roles within health care [2]. CEOs deal with demanding scenarios like long, difficult work hours, separation from loved ones, severe mental health issues, and battling false online narratives and campaigns by social media [3]. It was reported that almost a third (29%) of all the CEOs were forced out from their roles due to poor performance, a scandal or a strategic disagreement. Further, 7% of executives were formally dismissed and the remaining 66% quit with no reasons highlighted as such [4–7].

It has been highlighted that the increasing CEO turnover is a matter of concern for Australian and international health care organisations [8]. Deleterious consequences of CEO turnover include organisational unsteadiness, budgetary issues, loss of human resources and adverse effects on staff wellbeing and patient care [5]. Two thirds of health CEOs claimed that their decision to leave the organisation was solely their own [9]. Majority of the CEOs perceive their departure as detrimental towards their organisation i.e., community relations, medical staff relations, hospital culture and employee morale get negatively impacted. Interestingly, it was found that CEO’s turnover has a major knock-on effect on their executives. For example, the American College of Healthcare Executives (ACHE) report found that within one year of the CEO’s departure, the chief medical officer and the chief operating officer also changed, 77% and 52% of the time [10].

Over the years, organisations have realised the importance of succession planning as it enables an organization to sustain itself in their leader’s absence [1]. Organizations need to take seriously how they develop leaders because a strong leadership team can make a big difference on how health can be advanced for everybody [11]. In the health setting, it was noted that the average tenure of a CEO was about five years, and just over half (51%) had previously been a CEO at another facility [9]. In Australia, long-term employment contracts are considered for non-health settings to ensure that CEOs have sufficient time and outlook for decision making. Information on contract length among Australian CEOs is hard to gather, because not all organizations uncover the contract in their yearly report [12]. Among those Top 100 organizations that do uncover contract length stated that the contracts ranged between 3-and 5-years. Similarly, in the health setting, most CEO contract terms in USA are for three to four years and include provisions for renewal [13].

Two recent studies conducted in non-health settings have reported various reasons behind the turnover such as death, illness, dismissed for job performance, terminated for behavioural or policy-related problems, voluntary retirement, to pursue a new venture and departure following a merger or acquisition [13, 14]. However, in the health setting CEOs face different issues and challenges [5] hence may have various reasons to leave an organisation. The factors influencing CEO turnover in the healthcare sector differ significantly from those in non-health setting due to the industry’s unique organizational structures and compensation models. Healthcare organizations, whether for-profit or not-for-profit, operate within a complex regulatory environment and face distinct financial pressures related to reimbursement and healthcare policies [15]. Moreover, compensation structures for healthcare CEOs often include performance metrics related to quality of care and patient outcomes, which may not be as prominent in other industries [16]. These differences in setting underscore the importance of considering industry-specific factors when interpreting results on CEO turnover in healthcare, as they reflect the intricacies of managing healthcare delivery amidst regulatory, financial, and mission-driven demands. To date, no scoping reviews were published to identify the evidence associated with CEOs turnover. Detailing this evidence will clarify the gaps in this area and enable further research therefore, this scoping review aims to examine the evidence associated with CEO turnovers in both the health and non-health setting. Barriers and facilitators for CEOs retention were also identified. These finding will immensely help the health care industry in retaining their CEOs leading to lesser disruption in health services resulting in quality patient care.

## Method

The scoping review methodology followed the guidance published by Peters et al. 2020 [17].

### Inclusion criteria

#### Types of participants

Studies with CEO turnover in health and non-health settings are included. For the purpose of this study, CEOs were defined as the highest ranked officer within a registered corporate entity, such as a company or non-profit organisation. They usually report to a board of directors [18].

#### Concept

The factors or causes of CEO departure, barriers and facilitators contributing to CEO retention.

#### Context

The context of this review included both the health and non-health setting for comparative purposes. Examples of these included hospitals and corporate organisations.

#### Types of studies

Health care and non-health CEO turnover rates nationally and internationally were searched. Both quantitative and qualitative study designs were included in this scoping review. We also included systematic reviews to explore turnover and retention rates of CEOs by pearling of the reference list. Organisational reports through manual search, non-peer review literature was also included in the review. A protocol detailing the methodology for the current review was registered in Open science framework; Registration DOI- 10.17605/OSF.IO/8UXKH.

#### Search strategy

In consultation with a librarian, a three-step search strategy was utilised in this review. An initial limited search of Proquest (ABI/Inform), Medline (Ovid), CINAHL (Ebsco), Cochrane Library was undertaken which was followed by examining the text words contained in the title, abstract and of the index terms used to explain the article. Several studies were identified and deemed to be relevant to the review. A second search was conducted by identifying the keywords and index terms retrieved from our initial search was undertaken across all included databases. The below given databases were searched on 04 July 2021 using the support of a librarian: Proquest (select suitable relevant databases – e.g. ABI/Inform), Medline (Ovid), Pubmed, CINAHL (Ebsco), Cochrane Library, Informit databases, Scopus and Google Scholar. The above data bases were searched as they were anticipated to capture the relevant studies. In addition to the above, databases such as Web of Science, Business Source Ultimate and Emerald Insight were searched to identify the studies in non-health settings. All the above databases were explored during the final search. The search strategy of all the databases followed the same strategy mentioned in Appendix I. The reference lists of all identified reports and articles were searched for additional studies. We included literature in English in our review because of limited resources. Studies published from 1990 onward until 04 July 2021, were included as the CEO turnover issue has been identified since the changes in governmental funding mechanism for medical care [19]. Funding system has evolved over the past three decades, which has imposed a significant impact on the way that hospitals and other health organisations are managed [20]. The world economy and business models have experienced substantial transformation between 1990 and 2021, marked by the rise of globalization, the proliferation of digital technologies, and the emergence of new economic powers. These changes have reshaped industries, altered consumer behaviour, and necessitated adaptation to new market dynamics [20]. The following keywords were used to identify health studies: CEO, Chief executive officer, Vice-president, Top executive, Turnover, Replacement, Retention, Churn, Succession, Cause, Factors, Succession policy/policies, Health care, Hospital/hospitals. Appendix 1 details the search strategy used for health studies. A similar search was used for non-health studies by adding key terms such as firms, company, business, industry, organisation, enterprise and corporation.

### Exclusion criteria

Studies not identifying causes of CEO turnover were excluded. Publications in a language other than English were also excluded due to limited resources.

#### Data extraction

To address the review question, pertinent data were extracted from the included studies. To minimise the risk of bias and promote transparency, we registered and published the protocol before starting the review. Moreover, extraction was conducted by two authors to minimise the risk of errors and bias. When disagreement arose during data extraction conducted between authors, it was typically resolved through discussion, consultation with another senior researcher, or referral to established criteria, ensuring transparency and accuracy in the extraction process. The data extracted included the following: CEO turnover rates, causes, barriers, facilitators, authors, date of publication, country where study was originally conducted, aims/purpose, study population, methodology/methods, context, details, and key findings and outcome measures that relate to the scoping review question. The extracted data were represented in a logical format and three step coding process was followed to extract high level themes from the literature where the data was categorised into personal, organisational and performance related factors behind a CEO’s departure to enable a detailed map of the evidence [21].

## Results

**Fig. 1:**
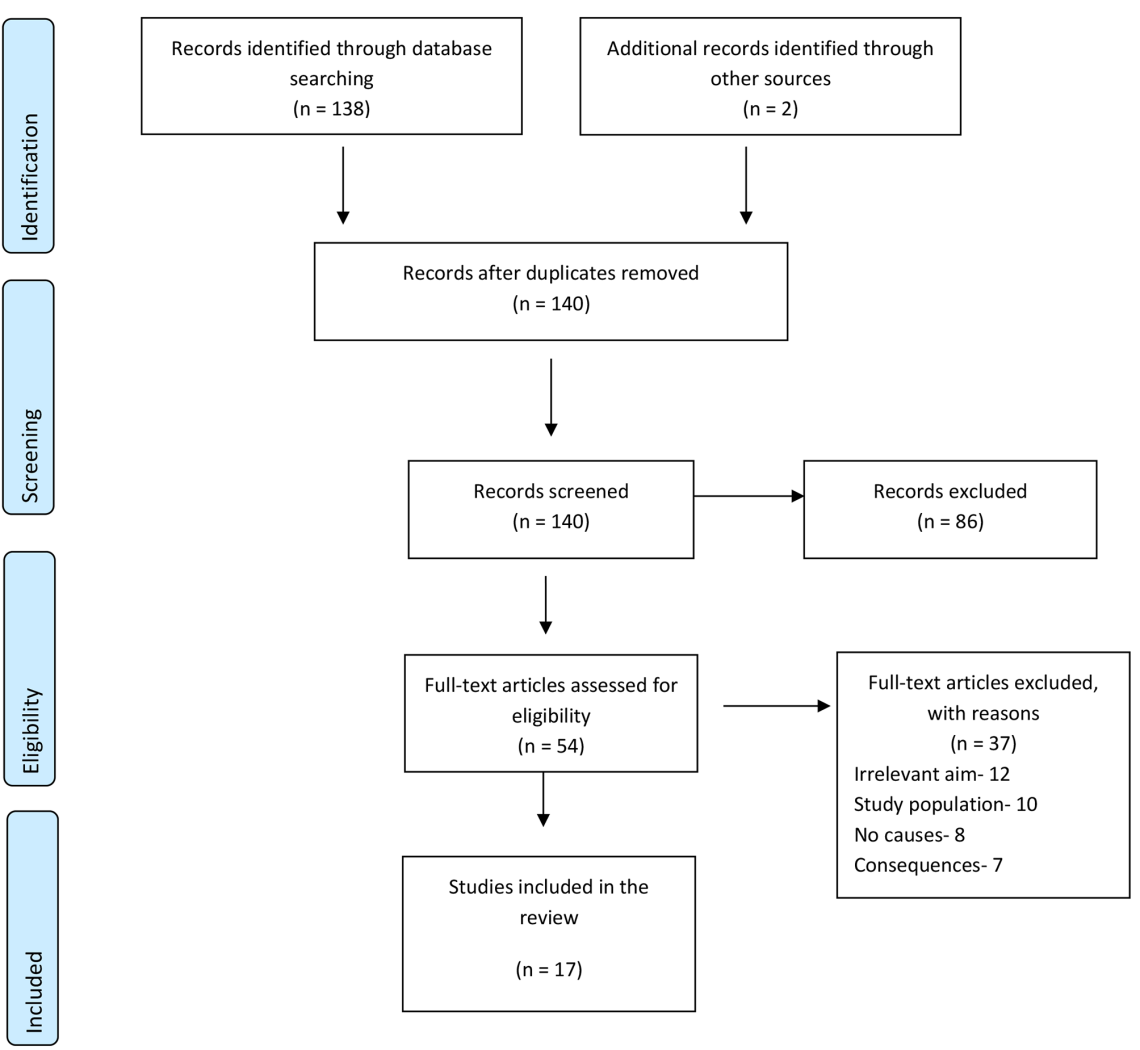
Prisma flowchart of study selection and inclusion process [22]

The database searches yielded a total of 138 citations (122 non-health and 16 health studies) after duplicates were removed and an additional two citations were found via hand searching (health studies). The titles and abstracts for these 140 citations were screened, with 86 papers excluded. The remaining 54 citations were considered for further detailed assessment of the full paper, and 37 were excluded due to having irrelevant aims (12 studies), study population (i.e., not relating to CEOs) (10 studies), not identifying cause behind CEO turnover (8 studies), and describing only facilitators of CEO turnover (7 studies). The search yielded a total of 17 citations for inclusion in this review i.e., 13 health and 4 non-health studies were included. A protocol detailing the methodology for the current review was followed. A flowchart showing the number of citations at each stage is detailed in Fig. 1: PRISMA flowchart of study selection and inclusion process.

**Table 1:** Characteristics of included studies

Studies/ Authors	Country where study was originally published	Aims/purpose	Study population	Methodology/methods	CEO turnover rates	Barriers	Facilitators	Context	Details/ Key findings	Tools used
**Health studies**										
Duffield et al.,2011 [5]	Australia	To identify the factors implicated in healthcare executive turnover in Australian public hospitals.	30 Health care executives	Quantitative Surveys were used in 11 hospitals across 3 Australian states participated.	16 executives left seven positions in 24 months which equates to 2.2 person in each role with a new incumbent every 6 weeks.	Education, lack of career advancement Opportunities, lack of remuneration, age and gender.	Appropriate challenges and opportunities for growth Clear succession planning in place	11 hospitals across 3 Australian states	Acting positions (46.34%) are common in Australian public hospitals.	Responses from the survey.
Khaliq et al.,2007 [9]	USA	To examine the general characteristics of CEOs, their hospitals and the perceived impact of CEO turnover on various organizational activities.	2118 Hospital CEOs.	Mail-based structured survey	1 out of every 6 hospital CEOs in the country either retires or moves on to a new position.	Most of the CEOs are forced out and some leave due to not being able to manage under-resourced smaller hospitals.	Not mentioned	916 high turnover and 3,130 low turnover hospitals.	Only about 21% of freestanding US hospitals undertake succession planning to prepare for turnover.	Responses from 23 survey question and SPSS 11.0.
Hearld et al.,2019 [11]	USA	To examine patterns of CEO change across US hospitals in different types of geographic locations.	Randomly selected 100 hospital CEOs	This study used data from 2 primary resources i.e., American Hospital Association (AHA) Annual Survey and Area Health Resources File for fiscal year 2005–2016.	Nearly one quarter of all U.S. hospitals experiencing a change in CEO every 3 to 4 years, on average.	Inadequate salary, desire for a better position elsewhere financial instability of the hospital, and market challenges	Leadership development programs and recruiting a leader from their own community may minimise turnover.	office based setting	Rural hospitals likely face substantial barriers to recruiting highly qualified CEOs	Univariate statistics provided summary characteristics of the hospitals.
Hospital CEO turnover rises, retirement up (USA). 2010 [23]	USA	To investigate the cause of CEO turnover among US hospitals.	US Hospital CEOs	Examining report of ACHE.	Hospital CEO turnover increased by 18% in 2009, the largest increase since 1999.	Poor financial performance or quality-of-care concerns and retirement	Not mentioned	Office based setting to examine ACHE reports.	Good succession planning is required to ensure organizational success	The ACHE report is based on changes in hospitals executive-level leadership
Weil et al., 1990 [19]	USA	To identify characteristics of hospitals experiencing rapid CEO turnover	US Hospital CEOs	Examining data available from American Hospital Association (AHA) between 1978 and 1988.	After the introduction of Medicare’s prospective payment system, the CEO turnover rates have accelerated.	Hospitals with low occupancy, low operating margins and lower costs per patient day	The organisations running on positive budgets may have longer CEO tenure.	Office based setting	Smaller hospitals in rural areas and not for profit hospitals experience higher CEO turnover.	Systematic review
Wilson et al.,2000 [1]	USA	To examine organizational factors contributing to hospital CEO turnover	US Hospital CEOs	Study examines organizational factors contributing to hospital CEO turnover.	Investor-owned hospital, are church related, offer many services, have high service usage	Larger hospitals with female CEOs are more likely to experience turnover may be due to family commitments.	Not mentioned	Not mention	some organizational environments are more conducive to turnover	Probit regression
Commins et al.,2014 [24]	USA	To examine the factors behind increasing CEO turnover.	US Hospital CEOs	Studying report released by ACHE	One in five hospital CEOs churned through the job in 2013.	Better positions; hospitals closure, hospitals consolidating leadership changes. demographic reasons and retirement.	Not mentioned	Office based setting	Importance of succession planning as frequent changes in leadership is not a good thing for hospitals.	Examining reports from ACHE
Leibert et al.,2010 [25]	USA	To identify various factors associated with small rural hospital CEO turnover	Small hospital CEOs from USA: (383 hospitals)	A structured survey was used consisting of closed and open-ended questions to collect the data on small rural hospital CEO turnover.	Hospitals had experienced more than 3 CEOs turnover during the previous 5 years	Poor communication between the CEO and the governing board. seeking a better career, getting out of an unstable health system,	CEOs having good interpersonal relationship with board members and medical staff	Small rural hospital of 5 states of USA	Succession planning is very important to minimise the CEO turnover	Responses received from the survey
Hart et al.,1993 [26]	USA	To examines rates of and reasons for turnover among administrators from 148 rural hospitals in four north-western states.	CEOs of 148 rural hospitals	Two separate surveys related to CEO turnovers were conducted–one of CEOs themselves and one of hospital board members.	During the study period, 85 CEO turnovers occurred at 78 hospitals.	Seeking a better position elsewhere, an unstable health care system, conflict with hospital board members, and inadequate salary.	Improving relationship with board members, getting adequate leadership and budgetary training.	148 rural hospitals in 4 northwestern states	Board members reported that they would not rehire their departed CEOs.	Standard two-tailed chi-square and t-tests are applied for statistical significance testing
Weil et al.,1995 [27]	USA	To examine the factors contributing to hospital CEOs voluntary decisions to leave their positions in 1990.	Random sample of 2,711 CEOs	A baseline survey was conducted of a random sample of 2,711 CEOs in the summer of 1989.	the year the prospective payment system was introduced, hospital CEO turnover was 12.8% and by 1988 it had risen to 18.4%.	Lower compensation. family-related obstacles such as spouse’s work or children’s school.	Variety in the job, Attractive pay and fringe benefits and the opportunity to advance career.	Short-term general medical and surgical hospitals	Voluntary turnover was found to be associated with higher levels of job dissatisfaction	A least-squares regression and logistic regression analysis.
Dwore et al.,1996 [28]	USA	A retrospective study to describe the rate and causes of CEO turnover between 1973 and 1987 in Utah community and non-community hospitals.	CEOs of Utah community and non-community hospitals	Researchers collected the data prospectively from 1988 to 1992 at each Utah community and non-community hospitals.	The annual average CEO turnover rate was 16.6%.	Factors of voluntary move includes: career moves, and job dissatisfaction. Involuntary moves: hospital market share decline, hospital terminated CEO’s, shorter tenure,	Hiring someone committed to the position, nurturing the CEO, providing adequate compensation,	Utah community and non-community hospitals	Certain factors in relation to turnover that are difficult to measure such as ambition, and influence of organisational culture.	The Pearson chi square statistics to analyse data and to compare data between periods.
Hudson et al.,1995 [29]	USA	To find out the career trajectories of ex-hospital CEOs.	4 Ex-Hospital CEOs	4 Ex-hospital CEOs shared their perspective in relation to the challenges of being health care CEO.	This paper informs the career trajectory of 4 ex-hospital CEOs.	CEOs informed that running a hospital can be extremely stressful and it can deprive one from family time and happiness.	CEOs tends to think that having good relationship with medical staff and board may help but it may not solve the issue.	N/A	Ex CEOs have started their own consultancy, and has to currently say that “I’m busy, not stressed.”	Responses from the interviews of 4 Ex-CEOs.
American College of health care Executive (2017) [4]	USA	Data examining the turnover rates of hospital CEOs in 2017	US Hospital CEOs	Examining the records of ACHE.	CEO turnover rate from 2013 to 2018 was 18% which declined to 17% by 2019.	It is a challenge to meet the health care demands as it continues to evolve and CEOs reaching retirement age.	Leadership development programs to support CEOs at their job.	ACHE report	Succession planning in place to minimise the negative impacts of turnover.	Data analysis of ACHE records
**Non- Health Studies**										
Coyne et al.,2007 [30]	USA	To investigate the turnover of CEOs in largest American firms.	More than 12 CEOs of largest American firms	Authors investigated the 2002–2004 CEO turnover rates of the top 1,000 U.S. companies and interviewed more than a dozen CEOs,	About 50% of all the largest firms will have a new CEO within 4 years.	CEO gets recruited from outside the company when the organisation is mid or low performing.	Not mentioned	Large American firms	CEO change is stressful for all the senior executives.	Compiling the database and responses from the interview.
Balsam et al.,2007 [31]	USA	To examine the association between equity compensation and voluntary executive turnover.	US Firm CEOs	Primary data source was S&P database which classified executive departure into four categories: (1) deceased; (2) retired; (3) resigned; and (4) unknown.	Not mention	Poor CEO performance, pressure from board. Equity compensation	Not mentioned	American firms	Higher the pay an executive is less likely to voluntarily leave the firm.	Descriptive statistics, regression analysis, Pearson correlation coefficient.
Kaplan et al.,2012 [32]	USA	To study CEO turnover – both internal and external– from 1992 to 2007 for a sample of large US companies.	US Firm CEOs	The sample runs from fiscal year-end 1991 to fiscal year-end 2007.	Since 2000, total CEO turnover increases to 16.8%, implying an average tenure of less than 6 years.	Stock performance, performance relative to industry, industry performance relative to the overall market and mergers and acquisitions.	Boards respond not only to poor performance relative to the industry but also to poor industry performance and to poor market performance.	American firms and office-based setting.	Majority of the turnover labelled as unforced is actually not voluntary.	Probit regressions estimating the probability of CEO turnover.
Gentry et al.,2021 [14]	USA	To introduce an open-source dataset documenting the reasons for CEO departure in S&P 1500 firms from 2000 through 2018.	S&P Firm CEOs	23 Coders were recruited to code the various causes of turnover events in last 19 years (2000–2018).	Initial search identified a total of 5,242 CEO departures over the 19-year period from 2000 through 2018 have occurred.	Death, illness, dismissed for job performance, terminated for behavioural or policy-related problems, Voluntary retirement.	Not mentioned	American firms and office-based setting.	Dismissal data is difficult to gather compared to turnover data.	Systematic review Descriptive statistics and pairwise correlations.

Abbreviation- American College of Healthcare Executives (ACHE), American Hospital Association (AHA)

### Date & country where study was origihnally published

Table 1 illustrates studies that were published between 1990 and 2021. All the studies described in the review were undertaken in developed countries. Of the studies, 94% were conducted in the USA, and 6% in Australia. There were no studies found in non-Western countries.

### Aim/Purpose

Most of the health studies (69%) included in this review aimed to investigate causes for the increasing turnover of CEOs with two studies reporting on turnover in rural hospital in USA [25, 26]. Only one study was identified from Australia that reported on health care executive turnover including CEOs. Two studies measured the organisational impacts of CEO departure [5]. Interestingly, a study explored the career trajectories of 4 CEOs i.e., what they are doing after leaving their CEO roles.

### Study population

One of the studies which was conducted in Australia focused on health care executive’s turnover in general. The health care executives are CEO, Chief Nursing officers (CNO), chief finance officer (CFO) etc. [5]. Other included studies focused specifically on CEO population. The population size for the included studies ranged from 4 to 2711 participants [26, 29].

### Study types

The types of studies ranged from Quantitative (23.53%), Qualitative research (11.76%) and report reviews (64.71%). Four of the health studies have used quantitative research to answer their research question [5, 9, 25, 27]. Two studies used a qualitative approach via conducting interviews [29, 30] to explore CEO’s perspective on roles, responsibilities and challenges of being a CEO therefore identified certain factors behind their turnover. The remaining studies reviewed the available data on CEO turnover from a hospital or health care college/organizations. On the other hand, one of the non-health studies have also used a qualitative approach to investigate the turnover rates [30]. The remaining three non-health studies reviewed the available data across various corporate organisations.

### CEO turnover rates

In Australia, 16 executives left seven positions in 24 months [5] whereas the USA is experiencing similar issues and turnover rates is expediting with average CEO tenure of 3 years. Non-health industry reports similar trajectory of CEO tenure of 4 years [14, 30, 31].

### Barriers

Health studies have identified certain factors behind turnover which ranged from lack of career advancement opportunities [4, 5], lack of remuneration [9], pressure from board [13], lack of engagement of teams within the organisation [14], job being demanding and stressful [4], poor financial performance [14], low occupancy in hospital [23], lack of leadership support programs [28], inadequate salary and desire for better positions [27].

Non-health studies highlighted some causes for their turnover such as poor financial performance [14], pressure from broad [30] and high job demands [32].

**Fig. 2:**
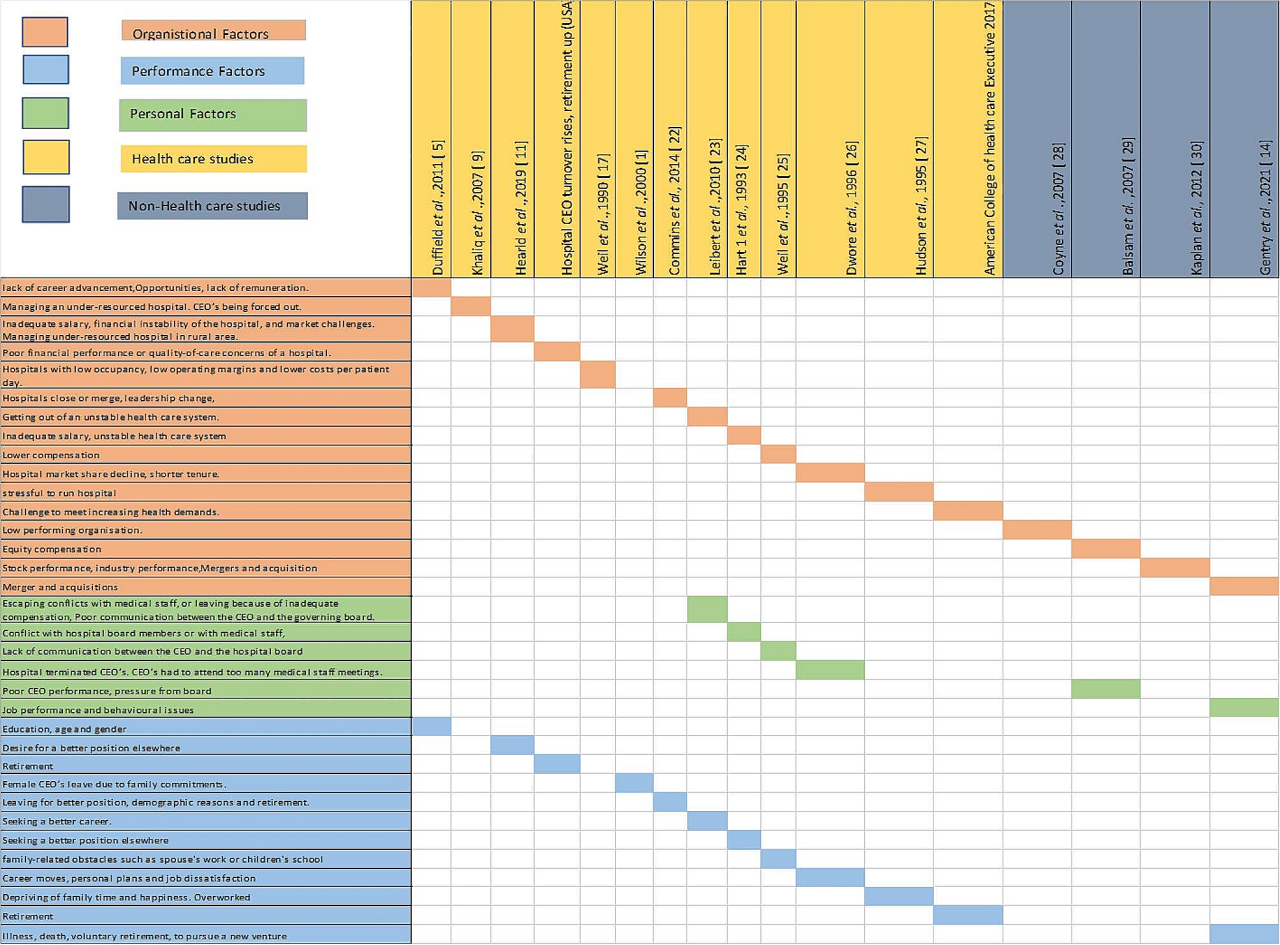
Classification of included health and non-health studies based on causes

Figure 2 details the classification of each of the studies. The studies ranged from having only one to all three of the components being organizational, performance and personal factors. The organisational component is aimed at focusing on organizational related causes due to which CEO leaves such as under-resourced hospitals, policy issues, merger and acquisitions. Performance components are aimed at the departure causes that are related to CEO’s unsatisfactory performance such as being terminated by the Board. Personal factors focus on CEO’s personal reasons behind resigning from their job such as family commitments, desiring a career change etc.

Sixteen studies have reported that CEOs depart due to organisational factors. Some of the reason’s identified behind their departure were poor financial performance, under-resourced services, low bed occupancy etc. [9, 14, 23]. CEOs have significant pressure in relation to finance and resource management, particularly if a hospital is under-resourced and has low bed occupancy rate which places more pressure on CEOs [8].

The second most common reason identified by the review behind their departure is due to their own personal reasons such as desiring for a career change, retirement, illness etc. [4, 14, 23, 24]. It is important to note that CEOs may desire for a career change or be willing to step down from their role for family commitments due to an increase in work demands [27, 29].

Six of the studies shows that CEOs contracts get terminated by the board due to their poor performance in not being able to meet the target goals [14, 25–27, 31]. Some other reasons include lack of leadership, conflict with board members or medical staff, not aligning the goals with organisational vision, ethical misconduct etc. [14, 26].

### Facilitators

Health studies have identified that certain strategies may help minimise the turnover such as supporting CEOs by exposing them to leadership development programs to enhance team engagement [4, 11]. Exposure to leadership programs will help them to reflect upon their own leadership style and learn new skills and knowledge in terms of managing their organisation/team. Moreover, CEOs get some time to detach themselves from the busy environment to meet with other CEOs/leaders from other organisation and share experiences [4]. The review suggests that developing good relationships with the board and its members may help in retaining a CEO as he/she may feel well supported [25–29]. Boards plays a vital role in supporting their CEOs as they are expected to intervene at the government level, where CEOs may not have the power or authority to do this [27]. It is also imperative for the CEO to have a good relationship with board chair so that the CEO can discuss issues concerning their organisation and get support as they need [28, 29]. Other factors to minimise turnover were variety in the job, good pay, good fringe benefits and the opportunity for professional development. CEOs also appreciate variety in their role where they get the opportunity to act in another CEO related role. They also feel valued if their efforts or achievements have been recognised and acknowledged by receiving incentives or fringe benefits [27].

## Discussion

The current scoping review consists of 13 health and 4 non-health related studies published over 31-year period that investigated and described the increasing CEO turnover rates. This review identified multiple factors associated with CEOs turnover in both health and non-health settings which is reported by the organisation. These included organisational, performance and personal.

A range of outcomes were measured in this review; they were the rate of turnover, causes behind turnover and consequences of turnover. Only one study identified the career trajectories of Ex-CEO’s. Regardless of health or non-health background, most of the studies have suggested the need for having a succession plan in place within their organisation [4, 5, 9, 23–25]. Researchers reports that an organisation performance suffers less from a CEO departure if they give other executive directors an opportunity to act in the CEO’s role when they are on leave or seconded to manage another organisation. Therefore, sudden CEO’s departure will be less impactful for the organisation as one of their own senior leaders will backfill the position until the recruitment process finishes, and a new CEO takes over [4].

The recruitment process in hospitals for CEOs varies, but many institutions are actively seeking capable and skilled leaders who possess a blend of healthcare expertise, strategic vision, and managerial acumen. However, challenges such as competitive market demands, complex regulatory environments, and the evolving landscape of healthcare delivery can impact the attractiveness of these positions to top-tier candidates [15, 24]. In Australia, long term contracts are considered for CEO’s in non-health settings however the downside to these contracts is a large termination pay-out which could be salary for the entire remaining term of the contract. Nonetheless, a much bigger cost which is mostly overlooked is the implication for all other layers of management within the organisation [12]. While long term contracts relieve the issue of CEO’s short-term decision making, it limits the prospects of senior executives aspiring for a CEO role thus, they end up leaving the organisation [12]. This review has identified similar trajectory of CEO turnover in both health and non-health settings which is due to similar reasons such as unsatisfactory CEO performance and personal factors such as death and illness. It has been noted that CEO’s left mainly due to performance and personal factors within non-health setting when compared to organisational factors in the health setting. Apart from that, both settings have highlighted the importance of the board in CEO retention. Other factors such as investing in leadership programs and providing incentives to the CEO are some of the measures that may help in CEO retention. Moreover, having some realistic and clear key performance indicators (KPIs) will be a facilitator for CEO retention [14]. One of the reasons why CEO’s may fail to perform well in their role is that the board may not have set clear KPIs for them to achieve. Therefore, if not addressed, this may result in poor performance [31]. Research shows that boards of directors play a significant role in CEO retention by implementing effective governance practices and strategic oversight, thereby influencing CEO turnover rates [35]. By adopting measures such as succession planning, performance evaluations, and incentivizing long-term organizational goals, boards can mitigate CEO turnover and enhance stability within the executive leadership [36]. Hospital CEO’s come from a wide variety of backgrounds and college education. Generally, Licensing and certifications are not required however having clinical knowledge and commitment to the profession can be helpful in advancing toward becoming a hospital CEO [33, 34]. One of the retired hospital CEO stated that starting his career as a clinician has immensely helped in gaining the respect that he developed while working alongside other medical professionals which was critical to his success as CEO [33].

Further research is required to identify CEOs turnover and which interventions are likely to increase their retentions. Developing a retention model for this group of employees has the potential to enhance both recruitment and is likely to stabilise the turnover rates for this population.

### Limitations of this review

The results described here are difficult to compare as both the health and non-health settings and structure of organisations are quite different. Additional limitation to the review was the language restriction as we only included studies published in the English Language as well as the how old some of the included studies were, spanning over a 31-year period. This review does not include an assessment of the quality of evidence, in keeping with a scoping review.

## Conclusion

This review has identified multiple causes for the CEOs departure which has been classified under the following components as: Organisational factors, performance factors and personal factors. More studies are needed to explore the reasons behind CEO’s departure so that retention strategies can be put in place to support CEOs at workplace. To minimize CEO turnover in hospitals, strategies includes fostering a supportive organizational culture that values transparency, employee development, and work-life balance, while also implementing robust succession planning programs to ensure smooth transitions and continuity in leadership.

### Electronic supplementary material

Below is the link to the electronic supplementary material.

Supplementary Material 1

## Data Availability

The authors confirm that the data supporting the findings of this study are available within the article [and/or] its supplementary materials.
