# Supplementary material for: Factors associated with health CEO turnover - a scoping review
Source: BMC Health Serv Res. 2024 Jul 29;24:861. doi: 10.1186/s12913-024-11246-y (PMC11288093; doi:10.1186/s12913-024-11246-y)
Supplement: Supplementary file 1 — Supplementary Material 1 [file 12913_2024_11246_MOESM1_ESM.docx]

**Appendix 1**

| [Set](https://search-proquest-com.ez.library.latrobe.edu.au/recentsearches.recentsearchtabview.recentsearchesgridview:toggellistorder?t:ac=RecentSearches) | Search | Databases | Search notes | Results |
| --- | --- | --- | --- | --- |
| S17 | ti("The consequences of executive turnover") | 5 databases | Checking to see if “Gold standard” paper is in the databases (it’s not available so we can’t test the search) | 0 |
| S16 | noft(("chief executive officer" OR ceo OR vice-president OR "executive director" OR "managing director")) AND noft((turnover OR retention OR replace OR replacement) NEAR/3 (cause OR causes OR factor* OR reason*)) AND noft((health care*)) | 5 databases | Adding some concept 4 terms to S5 | 11 |
| S15 | noft(("chief executive officer" OR ceo OR vice-president OR "executive director" OR "managing director")) AND noft((turnover OR retention OR replace OR replacement) NEAR/3 (cause OR causes OR factor* OR reason*)) | 5 databases | Testing search in S14 with additional business databases – does not increase results by much | 28 |
| S14 | noft(("chief executive officer" OR ceo OR vice-president OR "executive director" OR "managing director")) AND noft((turnover OR retention OR replace OR replacement) NEAR/3 (cause OR causes OR factor* OR reason*))Limits applied | ABI/INFORM Collection | Limiting S14 to scholarly publications. These results look rather low – likely to miss relevant papers | 26 |
| S13 | noft(("chief executive officer" OR ceo OR vice-president OR "executive director" OR "managing director")) AND noft((turnover OR retention OR replace OR replacement) NEAR/3 (cause OR causes OR factor* OR reason*)) | ABI/INFORM Collection | Combining CEO terms AND (turnover terms *NEAR* cause terms) | 183 |
| S12 | noft((turnover OR retention OR replace OR replacement) NEAR/3 (cause OR causes OR factor* OR reason*)) | ABI/INFORM Collection | Testing using proximity for Concept 2 and Concept 3 (ie for ‘turnover’ words near ‘cause’ words) to see if this gives more useful results | 3,927 |
| S11 | noft(("chief executive officer" OR ceo OR vice-president OR "executive director" OR "managing director") ) | ABI/INFORM Collection |  | 3,257,157 |
| S10 | (noft(("chief executive officer" OR ceo OR vice-president OR "executive director" OR "managing director") NEAR/3 turnover) OR noft(("chief executive officer" OR ceo OR vice-president OR "executive director" OR "managing director") NEAR/3 retention)) AND noft(cause OR causes OR factor* OR reason*) | ABI/INFORM Collection | Combining S3 AND S9 | 202 |
| S9 | noft(cause or causes or factor* or reason*) | ABI/INFORM Collection |  | 3,054,412 |
| S8 | noft(("chief executive officer" OR ceo OR vice-president OR "executive director" OR "managing director") NEAR/3 turnover) OR noft(("chief executive officer" OR ceo OR vice-president OR "executive director" OR "managing director") NEAR/3 retention) AND noft((causes OR reason OR reasons OR factor OR factors))Limits applied | ABI/INFORM Collection | Limiting S7 to scholarly publications – not sure if this was actually searching correctly therefore redid this in following searches | 419 |
| S7 | noft(("chief executive officer" OR ceo OR vice-president OR "executive director" OR "managing director") NEAR/3 turnover) OR noft(("chief executive officer" OR ceo OR vice-president OR "executive director" OR "managing director") NEAR/3 retention) AND noft((causes OR reason OR reasons OR factor OR factors)) | ABI/INFORM Collection | Including some additional Concept 3 terms | 1,166 |
| S6 | noft(("chief executive officer" OR ceo OR vice-president OR "executive director" OR "managing director") NEAR/3 turnover) OR noft(("chief executive officer" OR ceo OR vice-president OR "executive director" OR "managing director") NEAR/3 retention) AND noft(causes or reason or reasons)Limits applied | ABI/INFORM Collection | Limiting S5 to scholarly publications | 417 |
| S5 | noft(("chief executive officer" OR ceo OR vice-president OR "executive director" OR "managing director") NEAR/3 turnover) OR noft(("chief executive officer" OR ceo OR vice-president OR "executive director" OR "managing director") NEAR/3 retention) AND noft(causes or reason or reasons) | ABI/INFORM Collection | Adding some concept 3 terms | 1,153 |
| S4 | noft(("chief executive officer" OR ceo OR vice-president OR "executive director" OR "managing director") NEAR/3 turnover) OR noft(("chief executive officer" OR ceo OR vice-president OR "executive director" OR "managing director") NEAR/3 retention)Limits applied | ABI/INFORM Collection | Limited S3 to scholarly publications | 446 |
| S3 | noft(("chief executive officer" OR ceo OR vice-president OR "executive director" OR "managing director") NEAR/3 turnover) OR noft(("chief executive officer" OR ceo OR vice-president OR "executive director" OR "managing director") NEAR/3 retention) | ABI/INFORM Collection | Trialling use of proximity operator (NEAR/n) – ie searching for terms within “n” words of each other, in any order | 1,458 |
| S2 | noft(("chief executive officer" OR ceo OR vice-president OR "executive director" OR "managing director")) AND noft((turnover OR retention))Limits applied | ABI/INFORM Collection | Limited to scholarly publications | 976 |
| S1 | noft(("chief executive officer" OR ceo OR vice-president OR "executive director" OR "managing director")) AND noft((turnover OR retention)) | ABI/INFORM Collection | Searching just ABI/Inform for Concept 1 AND Concept 2 terms | 19,497 |
